# Intensity of Resistance Exercise and Its Effects on Pain, Functionality, and Quality of Life in Adults with Fibromyalgia: A Systematic Review

**DOI:** 10.3390/jfmk10020121

**Published:** 2025-04-05

**Authors:** Kevin Paúl Guachizaca Moreno, Lucía Fernanda Flores-Santy, Israel Vinueza Fernández

**Affiliations:** 1 School of Physical Therapy, Pontificia Universidad Católica del Ecuador, Quito 170143, Ecuador; kpguachizaca@puce.edu.ec; 2 MOVS Research Group, Pontificia Universidad Católica del Ecuador, Quito 170143, Ecuador; 3 Research Center for Health in Latin America (CISeAL), School of Physical Therapy, Pontificia Universidad Católica del Ecuador, Quito 170143, Ecuador; iavinuezaf@puce.edu.ec

**Keywords:** fibromyalgia, exercise, resistance training, pain, quality of life

## Abstract

Background: Fibromyalgia is a chronic rheumatological disease that affects the musculoskeletal system, primarily characterized by widespread chronic pain and other symptoms that significantly impact the quality of life of those who suffer from it, being more prevalent in the female population. In this context, among the non-pharmacological treatments available for this condition, resistance exercise has shown to be a promising intervention. The aim of this systematic review was to determine the optimal intensity of resistance exercise in patients with fibromyalgia and evaluate its effects and benefits. Methods: An exhaustive literature search was carried out in the PubMed, Scopus, SciELO, Web of Science, ScienceDirect, and PEDro databases. After the selection process, from the 405 studies initially identified, 17 met the established inclusion criteria. Subsequently, the methodological quality of the studies was evaluated using the Cochrane RoB 2 tool. Results: From the 405 studies initially identified, 17 met the established inclusion criteria. The results indicate that among the interventions studied, progressive intensity is the most recommended, where it is suggested to start with 40% of 1RM. This is followed, in order of recommendation, by low- and medium-intensity exercises, while high-intensity exercises are, in principle, the least used. Furthermore, the analysis of the effects of progressive intensity exercise showed promising results, including a significant decrease in pain, an increase in physical functionality, and, consequently, an improvement in quality of life. Conclusions: These findings suggest that progressive resistance exercise is an effective intervention for treating patients with fibromyalgia.

## 1. Introduction

Fibromyalgia (FM) manifests as a complex rheumatic condition affecting the musculoskeletal system, causing chronic and widespread pain [1]. It is often squired by additional symptoms, including chronic fatigue, muscle stiffness, digestive issues, sleep disturbances, and mood changes [2]. This condition predominantly affects women, with a prevalence of 75% to 90% of the cases, and is characterized by abnormal pain processing, marked by an excess of excitatory neurotransmitters, particularly substance P, which is found at 2 to 3 times higher levels in cerebrospinal fluid than in individuals without the condition [3]. Elevated levels of glutamate are also present, along with hormonal, sleep, cognitive, and autonomic nervous system dysfunctions [4]. Although a specific cause is not always identified, genetic, environmental, and hormonal factors and certain infections can influence the condition. The multifaceted nature of FM requires a comprehensive treatment approach that combines pharmacological and non-pharmacological interventions [5]. Exercise is a promising non-pharmacological approach for managing FM [6]. Among various exercise modalities, strength or resistance exercise (RE) has proven to be particularly effective in improving FM symptoms [7]. This exercise involves muscle contractions against external resistance, utilizing weights, resistance bands, machines, or body weight [8]. RE has enhanced muscular strength, power, endurance, and mass. Numerous clinical studies have demonstrated its efficacy in improving the overall condition of FM patients [9].

Nowadays, research focuses on the impact of resistance training at different intensity levels on the symptoms experienced by people with FM. These intensity levels include high-intensity (HIRE), medium-intensity (MIRE), low-intensity (LIRE), and progressive-intensity (PIRE) protocols. Several methods prescribe and monitor resistance training intensity, each with specific thresholds for different intensity levels [10]. One standard method uses the percentage of 1 repetition maximum (1RM); in this approach, HIRE is typically defined as ≥80% of 1RM or <15 repetitions to failure, whereas LIRE involves <60% of 1RM or >15 repetitions to failure [11]. Another modality is the Borg scale (6–20), which categorizes HIRE as ≥15, moderate intensity as 12–14, and LIRE as <12 [12]. The maximum voluntary capacity (MVC) method also defines HIRE as usually ≥80% of MVC, moderate intensity as 50–79% of MVC, and LIRE as <50% of MVC [13]. Furthermore, the OMNI Generalized Subjective Exertion Scale (OMNI-GSE) (0–10) considers HIRE to be ≥7, moderate to be 4–6, and low to be <4 [14]. These measures allow patients, researchers, and healthcare providers to accurately monitor resistance training intensities across different protocols and individual capacities. This exercise modality is used for various reasons, including athletic performance enhancement, aesthetic improvements, rehabilitation, and general health maintenance [15]. Currently, the potential benefits of RE for FM patients are multiple and significant [16]. RE can improve pain, physical functionality, and quality of life (QOL), which are the factors that will be analyzed in this research and that are commonly affected in FM patients [17]. Thus, RE can be seen as a promising intervention for the comprehensive management of this complex condition.

The literature on RE has increased significantly in recent years for many reasons. This increase is due to their efficacy as a therapeutic tool for treating FM and their benefits to overall well-being [18]. However, researchers are still trying to determine the optimal intensity to mitigate the adverse effects caused by FM [19]. Systematic reviews have focused on the general effects of RE in FM. However, the optimal intensity of RE to control FM symptoms remains debated in the scientific and medical community [20]. In patients with FM, chronic widespread pain represents the main symptom, significantly affecting their ability to perform daily activities. Physical functionality is severely compromised, limiting mobility and increasing dependency, while quality of life decreases notably due to the combination of persistent pain, sleep disturbances, chronic fatigue, and associated psychological symptoms. Therefore, this systematic review aims to analyze the effects of RE at various intensities on pain, functionality, and QOL in adults with FM.

## 2. Study Design

The methodology of this systematic review adhered to the Preferred Reporting Items for Systematic Reviews and Meta-Analyses (PRISMA) guidelines [21] and was registered in PROSPERO (CRD42024580390). According to this registered protocol, the systematic search was designed to be completed by August 2024. A modification to the original title was made to be more specific in the description of the study topic, allowing for a more comprehensive analysis of resistance training interventions as found in the literature. This modification solely sought to clarify the terminological scope of the study, while all the originally established methodological parameters were maintained without alteration.

### 2.1. Search Strategy

The search was carried out in the following electronic databases: PubMed (National Library of Medicine and National Institutes of Health), Scopus, SciELO (Scientific Electronic Library Online), Web of Science, ScienceDirect, and PEDro (Physiotherapy Evidence Database). The final search was completed in August 2024, considering the entire period of the electronic databases.

Our search strategy combined terms related to the condition and intervention using appropriate Boolean operators. The core search structure across all the databases included variations in “fibromyalgia” combined with exercise-related terms such as “resistance training”, “strength training”, and “resistance exercise.” For example, in PubMed, the following search string was used: “fibromyalgia” AND (“resistance training” OR “Resistance Training” OR “strength training” OR “strength training” OR “resistance exercise”). Similar search structures were adapted for other databases according to their specific indexing requirements and search capabilities. Database-specific filters for clinical trials and human studies were applied where available. The complete detailed search strategy for each database, including all the specific terms, filters, and combinations used, can be found in Appendix A.

### 2.2. Eligibility Criteria

The selection criteria included clinical trials in English or Spanish examining adults (≥18 years) of both sexes with FM diagnosis according to the American College of Rheumatology criteria. Studies evaluating the effects of RE programs lasting more than two weeks were considered, explicitly analyzing whether there are benefits or superiority in working with different intensities on the QOL of people with FM.

Eligible studies had to include a control group with the same FM pathology or healthy people, subjected to different intensities of RE. The effects on various aspects were evaluated, including the main ones: pain, functionality, and QOL of people with FM.

The following exclusion criteria were adopted: case studies, theses, and dissertations; RE programs without quantifiable intensity specification, as the primary objective of this review was to compare effects of different resistance training intensities, making studies without clearly defined and measurable intensity parameters impossible to classify into comparable groups and therefore unsuitable for addressing our central research question; other exercise modalities such as yoga, tai chi, or pilates; groups without exercise intervention; and studies using drugs as the sole treatment.

For the classification of resistance exercise intensity, standardized criteria were applied (high intensity ≥80% of 1RM [11], medium intensity 60–79% of 1RM, low intensity <60% of 1RM [11], Borg scale (6–20) [12], maximum voluntary capacity [13], and OMNI-GSE (0–10) [14]). The studies were categorized based on the information provided by the original authors.

Here is the complete PICOS framework text in English with the addition of experimental studies:

Inclusion criteria were defined using the PICOS framework:

- Population (P): Adults (≥18 years) of both sexes with fibromyalgia diagnosis according to the American College of Rheumatology criteria.
- Intervention (I): Resistance exercise programs lasting more than two weeks with quantifiable intensity specifications.
- Comparison (C): Control group with the same fibromyalgia pathology or healthy people subjected to different intensities of resistance exercise.
- Outcomes (O): Effects on pain, functionality, and quality of life in people with fibromyalgia.
- Study design (S): Randomized controlled trials, controlled clinical trials, and experimental studies published in English or Spanish.

### 2.3. Data Extraction

Data extraction was performed by three experienced researchers (KPGM, ISVF, and LFFS). Two independent researchers (KPGM and ISVF) selected the articles using the Rayyan CRQI tool [22] and subsequently extracted the data. This platform allowed for a blinded selection process, eliminating potential biases, as each reviewer validated the information independently without knowing the other’s decisions. Once the individual assessment was completed, the blinding was removed to identify disagreements. The data were extracted using a standardized form designed to systematically collect relevant information from the included studies. The third experienced researcher (LFFS) intervened in cases of discrepancy during both the selection and data extraction phases, facilitating discussion until consensus was reached. When addressing missing data in the primary studies, instances were documented as ’not reported’ and considered during the risk of bias assessment and in the interpretation of results. The selection process followed a systematic order: first, the article titles were reviewed, followed by an evaluation of the abstracts, and finally, for potentially eligible articles, the full texts were analyzed.

A structured data extraction procedure was conducted to document the following: study characteristics (design and authors), participant demographics (sample size, age, and sex distribution), intervention details (exercise modality, duration, and intensity parameters), adherence rates, and primary outcomes. The methodological quality of the selected studies was assessed using the Cochrane Risk of Bias Tool to evaluate potential sources of bias [23].

Several critical considerations regarding data synthesis and analysis guided our methodological decisions. We opted not to conduct a meta-analysis due to the substantial clinical heterogeneity across the included studies. This heterogeneity manifested in multiple dimensions: variability in resistance exercise protocols (intensity, frequency, and duration); diverse outcome measurement tools; differences in participant characteristics (disease duration and symptom severity); and inconsistent control and comparison groups. Quantitative assessment using I^2^ statistics reinforced this decision, revealing values consistently exceeding 75%, indicating high statistical heterogeneity.

Under these circumstances, pooling the data could yield misleading results that may result in inappropriate clinical recommendations. Instead, we adopted a qualitative synthesis approach designed to embrace this heterogeneity, facilitating a more nuanced interpretation of the evidence while acknowledging the methodological limitations of individual studies.

In accordance with our protocol, we analyzed the data solely based on the published information, without contacting the study authors for any missing data. This decision was driven by practical time limitations and concerns that inconsistent response patterns from the authors could introduce selection bias. Regarding publication bias, traditional assessment tools such as funnel plots were not suitable for our qualitative review. We addressed this limitation by employing a robust search strategy across diverse databases, including those from developing regions, with no restrictions on language or publication status. We explicitly acknowledged these methodological constraints when interpreting our findings.

## 3. Results

**Figure 1:**
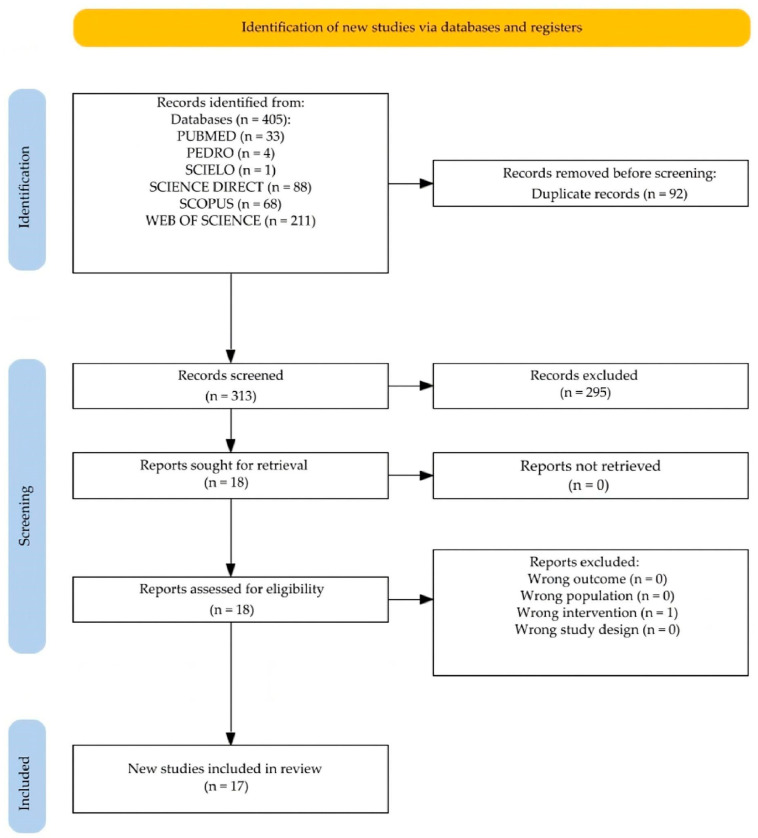
PRISMA flow diagram showing the systematic review selection process.

In the initial database search, 405 records were identified. After removing 92 duplicates and screening titles and abstracts, 74 articles were evaluated in full text. Finally, 17 studies that met the inclusion criteria were selected for final analysis (Figure 1).

### 3.1. Characteristics of Included Studies

Among the seventeen studies that were included in the review, the oldest publication was in 2001 [24], and the most recent was in 2022 [20,25]. The studies were conducted in Finland [24,26], South Korea [27], the USA [28,29], Spain [25,30], Sweden [31,32,33,34], and Brazil, which stand out as presenting the highest number of publications [20,35,36,37,38,39]. Additionally, all the studies were required to provide information regarding the intensity of RE for each intervention. These included studies were randomized controlled clinical trials, except for one quasi-experimental study [25]. The total sample of the included studies comprised 993 women with FM, aged between 18 and 70 years, with pain, functionality, and physical quality being the most studied variables.

### 3.2. Instruments Used to Quantify the Intensity of RE

Various methods were employed to analyze RE intensities. The most prevalent approaches included the percentage of 1 repetition maximum (1RM) [11], the Borg scale (6–20) [12], the maximum voluntary capacity (MVC) method [13], and the OMNI Subjective Generalized Exertion Scale (OMNI-GSE) (0–10) [14]. These methodologies offer distinct ranges for categorizing exercise intensity into high, moderate, or low levels, facilitating a comprehensive assessment of resistance training protocols across studies.

### 3.3. Questionnaires Used on Pain, Functionality, and QOL

Various pain assessment tools were used in the studies to evaluate pain intensity and its impact on fibromyalgia patients. The Visual Analog Scale (VAS) was the most frequently used measure, specifically used in ten studies [24,26,27,30,31,33,34,36,38,39]. The Fibromyalgia Impact Questionnaire (FIQ), specifically its pain subscale, was the only instrument in two studies to assess pain alongside other fibromyalgia symptoms [32,37]. Notably, a study utilized VAS and FIQ, providing a more comprehensive pain assessment [35]. Additionally, two combined the FIQ with tender point assessment, offering insights into pain impact and sensitivity [28,29]. The Brief Pain Inventory (BPI) was utilized in one study to provide a comprehensive evaluation of pain intensity and its interference with daily activities [25].

Additionally, we decided to evaluate functionality in patients with FM, although not all the studies included specific functionality measures. FIQ, particularly its functionality subscale, was utilized in four studies to assess functional capacity alongside other fibromyalgia symptoms [27,30,34,35]. The Health Assessment Questionnaire (HAQ) was employed to evaluate functional status [24]. One study used the Senior Fitness Test [25], while the other utilized the Continuous-Scale Physical Functional Performance (CS-PFP) test for a comprehensive assessment of physical functionality [29]. Interestingly, although the SF-36 is not primarily designed to evaluate functionality, three studies employed this tool [36,38,39]. The authors of these studies inferred improvements in functionality based on changes in SF-36 scores, despite it not being a direct measure of functionality.

QOL, the third variable analyzed, was evaluated using several instruments, including FIQ or its revised version (FIQR), the most frequently used measure, appearing in four studies [27,30,32,37]. The Short Form-36 Health Survey (SF-36) was also commonly utilized, featuring three studies [33,34,36]. Notably, three studies employed both the FIQ and SF-36 [35,38,39], providing a more comprehensive assessment of QOL by combining a disease-specific measure with a general health-related QOL instrument. Another study used a different approach, opting for the QOL Scale (QOLS) [28]. This diverse array of assessment tools, sometimes used in combination with single studies, allows for a multifaceted examination of QOL in FM patients across various investigations.

In addition to the main parameters evaluated, other studies focused on particular components for FM patients, such as mental health and sleep quality. Depression, a common comorbidity in FM, was assessed using specific scales such as the Beck Depression Inventory-Fast Screen (BDI-FS), a specific measure for depression, which was utilized in four studies [24,28,30,38]. The Hospital Anxiety and Depression Scale (HADS), which assesses both anxiety and depression, was employed in four studies [32,33,34,37]. Interestingly, several studies used scales that are not specifically designed to measure depression, but from which the authors inferred improvements in depressive symptoms. For instance, FIQ was used in two studies [27,35], while the SF-36 Mental Health domain was employed in two others [36,39]. Although these scales primarily assess the overall impact of FM and general mental health, respectively, the authors interpreted improvements in these scores as indicative of reduced depressive symptoms. Similarly, one used the State-Trait Anxiety Inventory (STAI-S) [25], and one employed the Brunel Mood Scale (BRUMS) [20], both of which, while not depression-specific, were used to infer changes in mood and depressive states. Sleep quality, another critical factor in FM management, was evaluated using the Pittsburgh Sleep Quality Index (PSQI) [31,37]. The tools used are shown in Table 1.

### 3.4. Characteristics of Interventions

The analysis of training protocols revealed that most studies established a frequency of two sessions per week, with only one investigation implementing three sessions per week [39]. Regarding the temporal extension of these interventions, the programs varied from 2 to 24 weeks in duration.

The intervention protocols included four different RE modalities determined by the intensity of each (HIRE, MIRE, LIRE, and PIRE). RE was the main component, lasting 30 to 60 min. Each modality was applied according to the intensity assigned to the patients. Of the seventeen selected studies, one used HIRE [31], two employed MIRE [27,39], two opted for LIRE [28,30], and twelve implemented progressive intensity [20,24,25,26,29,32,33,34,35,36,37,38], with the latter being the most common due to its adaptability and preference among participants. However, the study protocols include various combinations of therapies. One integrated warm-up, stretching, and cool-down [20,24,25,27,28,29,30,31]. Another alternative combined warm-up and cool-down [25], while a different variant involved warm-up and stretching [32,37,38]. Additionally, an option consisting of only a warm-up was considered [33,34]. On the other hand, an approach focused solely on stretching [35]. Finally, a last approach comprised exclusively of the main RE [26,39]. Inter-set rest periods were only documented in a subset of studies, which consistently reported 60 s intervals. Detailed information about the intervention protocols, including rest intervals, total training volume, and progression schemes, is thoroughly presented in Table 2.

### 3.5. Effects of Resistance Training on Pain, Functionality, and QOL

When analyzing the effects of exercise on FM symptoms, PIRE exercise, evaluated in 12 studies, showed varied results across different parameters. Regarding pain, eight studies reported significant improvements [24,25,33,34,35,36,37,38], while three observed no changes [26,29,32]. One study did not evaluate this parameter [20]. Physical functionality improved significantly in six studies [24,29,34,35,36,38] but was not assessed in the remaining five [25,26,32,33,37].

Regarding QOL, five studies reported significant improvements [33,35,36,37,38], two observed no changes [32,34], and the remaining five did not evaluate this parameter [20,24,25,26,29]. Depression improved significantly in five studies [24,35,36,37,38], while five observed no changes [20,25,32,33,34]. Two studies did not evaluate depression [26,29]. Finally, sleep quality was assessed only in two studies, reporting significant improvements [25,37].

Moving on to LIRE, more consistent results were observed. Two studies reported significant improvements in pain and depression [28,30]. Physical functionality and QOL showed substantial improvements in one study [30], while others did not evaluate functionality and observed no changes in QOL [28]. Neither of these studies assessed sleep quality.

Regarding MIRE exercise, the two studies that evaluated it reported significant improvements in pain and physical functionality [27,39]. QOL and depression showed significant improvements in one study [39], while in the other no changes in these parameters [27]. As with the LIRE studies, sleep quality was not evaluated in either of these studies.

Finally, the only study that evaluated HIRE exercise showed mixed results. No significant changes were observed in pain, but significant improvements in sleep quality. Physical functionality, depression, and QOL were not assessed in this study [31]. The analyzed results are shown in Table 3.

### 3.6. Quality of Studies and Risk of Bias

**Figure 2:**
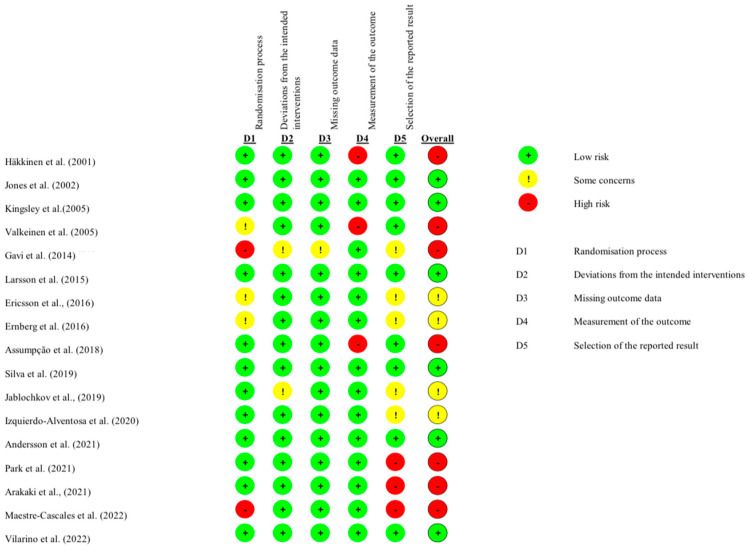
Risk of bias assessment for included studies based on Cochrane Collaboration’s tool [20,24,25,26,27,28,29,30,31,32,33,34,35,36,37,38,39].

The risk of bias assessment using the Cochrane RoB 2 tool revealed several methodological patterns across the 17 included studies. Selection bias was generally well controlled (82.4% of the studies), while performance bias was high due to the inherent difficulty of blinding participants to exercise interventions. Detection bias was minimized in 70.6% of the studies through blinded outcome assessment. Attrition bias was low in 76.5% of the studies, with appropriate handling of missing data. Reporting bias was consistently low across all the studies, with pre-specified outcomes adequately reported in Table 4 and Table 5, and Figure 2.

## 4. Discussion

This article aims to analyze the optimal intensity of RE in patients diagnosed with FM and investigate its effects and benefits. Our systematic review of the available literature from 2001 to 2024 identified 17 studies that met the established inclusion and exclusion criteria, allowing us to evaluate different intensity approaches and their impact on fibromyalgia symptoms. Throughout our analysis, we investigated how different RE intensity protocols influenced key outcomes, including pain, functionality, and quality of life in FM patients.

Historically, RE has been promoted as a conservative non-pharmacological alternative with quantifiable benefits for patients with FM [40]. However, its study has been limited by the belief that it increases pain, although current evidence demonstrates the opposite [41]. Thus, the results of the present study showed that 70.59% of the analyzed literature revealed a decrease in pain [24,25,27,28,30,33,34,35,36,37,38,39], along with other intervention benefits where 58.82% showed improvements in functionality [24,25,27,29,30,34,35,36,38,39], and 41.18% showed better quality of life [30,33,35,36,37,38,39]. A previous systematic review, conducted in 2021 with seven studies, presented similar results, although it highlighted an important limitation by focusing mainly on physical aspects, leaving aside the psychological aspects of the disease, concluding that while RE may be a useful treatment to address the clinical presentation of FM, several aspects and dosing still need to be explored [2].

Regarding the methodology employed, PIRE was the predominant method, representing 70.59% of the total analyzed studies [20,24,25,26,29,32,33,34,35,36,37,38]. The programs followed a specific pattern, beginning with low intensities distributed as follows: 41.67% started only at 40% of 1RM [26,32,33,34,37], 25% started at 40% to 60% of 1RM [24,29,38], 8.33% at 60% of 1RM [36], another 8.33% used a slightly intense on the Borg scale [35], 8.33% employed an OMNI scale of 3–4 [25], and 8.33% began with 2 sets of 12 repetitions [20]. This methodology aligns with a previous systematic review, where intervention protocols started at 40% 1RM intensity and increased gradually according to individual tolerance. The effectiveness of this personalized approach is evidenced by two key aspects: the high program completion rate by participants and the achievement of superior results compared to other modalities [42].

On their part, studies on LIRE, which constitute 11.76% of the analyzed literature [28,30], showed significant improvements in pain and depressive symptoms. A systematic review with meta-analysis on the dose–response of low-intensity activities, although not specifically resistance training, such as walking and yoga, suggests that the benefits are not limited to the physical component of exercise, but extend to biopsychosocial factors, including social interaction, reduction in negative thoughts through group activities, increased self-sufficiency, exposure to natural spaces, modifications in neurotransmitters, and perception of personal achievement. The tolerability and comfort of these interventions explain why participants maintained a constant intensity instead of progressing to more demanding levels, allowing for sustained benefits in mental health [43].

Regarding the next intensity level, studies on MIRE, representing 11.76% of the articles, showed decreased pain and increased functionality [27,39]. A recent meta-analysis revealed that moderate-intensity training (60–80% 1RM) significantly improves muscle strength, muscle mass, and functionality in patients with rheumatoid arthritis and osteoarthritis. This improvement in functionality is attributed to neuromuscular and structural adaptations, being relevant since most daily activities require moderate effort. However, in sedentary patients or those exercising for the first time, tolerance to moderate intensities may be initially limited, affecting adherence. Prescription should be individualized according to the patient’s clinical status, previous experience, and functional capacity [44].

Finally, in HIRE, only 5.88% of the analyzed studies implemented this level of training, observing only significant improvements in sleep quality, with no relevant changes in pain levels [31]. As a sample, a recent literature analysis suggests that high-intensity training (>80% 1RM) may generate positive adaptations in sleep patterns, possibly due to changes in hormonal regulation and muscle fatigue that favor the sleep–wake cycle. However, the limited available evidence reflects researchers’ caution in prescribing high-intensity exercises in FM patients, considering the risk of symptom exacerbation and potential low treatment adherence. Implementing high-intensity protocols requires a careful individualized assessment, considering each patient’s severity of symptoms, previous exercise experience, and pain tolerance threshold [45].

A significant limitation of this review is the lack of subgroup analyses based on age, sex, or baseline physical activity levels. Such analyses could reveal varied responses to different resistance exercise intensities across distinct patient populations, potentially enabling more personalized treatment approaches. Future research or meta-analyses incorporating these stratifications would help determine whether specific intensities are more effective for particular patient profiles, thereby enhancing the clinical applicability of exercise prescriptions for fibromyalgia.

## 5. Conclusions

Based on the analyzed scientific evidence, progressive RE emerges as the optimal intervention for patients with FM, establishing an initial recommendation of 40% 1RM. This therapeutic modality has not only proven to be the most studied, constituting 70.59% of the analyzed research, but has also demonstrated to be the most effective by systematically integrating the specific benefits of each intensity level: from pain reduction and improvement of depressive symptomatology at low intensities, progressing towards increased functionality at moderate intensities, up to achieving potential improvements in sleep quality at higher intensities.

## Figures and Tables

**Table 1:** Assessment tools and outcome measures used for evaluating pain, physical function, quality of life, sleep quality, and depression in patients with fibromyalgia.

Authors	Pain	Physical Functional Capacity	Quality Life	Sleep Quality	Depression
Häkkinen et al. [24]	VAS	HAQ	-	-	BDI-FS
Jones et al. [28]	TP, FIQ	-	QOLS	-	BDI-FS
Kingsley et al. [29]	TP, FIQ	CS-PFP	-	-	-
Valkeinen et al. [26]	VAS	-	-	-	-
Gavi et al. [38]	VAS	SF-36	FIQ, SF-36	-	BDI-FS
Larsson et al. [37]	FIQ	-	FIQ	PSQI	HADS
Ericsson et al. [32]	FIQ	-	FIQ	-	HADS
Ernberg et al. [33]	VAS	-	SF-36	-	HADS
Assumpção et al. [35]	VAS, FIQ	FIQ	FIQ, SF-36	-	FIQ
Silva et al. [36]	VAS	SF-36	SF-36	-	SF-36
Jablochkova et al. [34]	VAS	FIQ	SF-36	-	HADS
Izquierdo-Alventosa et al. [30]	VAS	FIQ	FIQR	-	BDI-FS
Andersson et al. [31]	VAS	-	-	PSQI	-
Park et al. [27]	VAS	FIQ	FIQ	-	FIQ
Arakaki et al. [39]	VAS	SF-36	FIQ, SF-36	-	SF-36
Maestre-Cascales et al. [25]	BPI	Senior Fitness Test	-	FIQ	STAI-S
Vilarino et al. [20]	-	-	-	-	BRUMS

Note: VAS = Visual Analog Scale; HAQ = Health Assessment Questionnaire; BDI-FS = Beck Depression Inventory-Fast Screen; TP = tender point assessment; FIQ = Fibromyalgia Impact Questionnaire; QOLS = Quality of Life Scale; CS-PFP = Continuous-Scale Physical Functional Performance; SF-36 = Short Form-36 Health Survey; PSQI = Pittsburgh Sleep Quality Index; HADS = Hospital Anxiety and Depression Scale; FIQR = Fibromyalgia Impact Questionnaire Revised; BPI = Brief Pain Inventory; STAI-S = State-Trait Anxiety Inventory; BRUMS = Brunel Mood Scale.

**Table 2:** Methodological characteristics of studies on resistance training at different intensities in patients with fibromyalgia.

Reference; Study	Type of Study	Sample Size	Age in Years	Intervention	Intensity
Häkkinen et al., 2001 [24]	RCT	RE: 11 CG: 10 HC: 12	RE: 39 ± 6 CG: 37 ± 5 HC: 37 ± 6	21 weeks: WF: 2 week **Exercises:** Isotonic exercises including supine, squats, extension, and flexion of knees and trunk.	**PIRE** Weeks 1–3: 1 set of 15–20 reps at 40–60% 1RM Weeks 4–7: 1 set of 10–12 reps at 60–70% 1RM Weeks 8–14: 1 set of 8–12 reps at 60–80% 1RM Weeks 15–21: 1 set of 5–10 reps at 70–80% 1RM
Jones et al., 2002 [28]	RCT	RE: 28 FG: 28	RE: 49.2 ± 6.36 FG: 46.4 ± 8.56	12 weeks: WF: 2 week **Exercises:** The resistance program used free weights and elastic bands, targeting three regions: trunk stabilizers (abdominal and paraspinal muscles), lower-limb muscles (ankle, knee, and hip muscle groups), and upper-body musculature (thoracic, scapular, and arm muscles). Specific exercises were not detailed.	**LIRE** Using hand weights (1–3 pounds) and resistance bands based on the patient’s tolerance, while staying aware of their body’s signals.
Kingsley et al., 2005 [29]	RCT	RE: 15 (8 completed the study) CG: 14 (12 completed the study)	RE: 45 ± 9 CG: 47 ± 4	12 weeks: WF: 2 week **Exercises:** The program included 11 isotonic exercises that targeted the following muscle groups: chest, leg extensors, leg flexors, shoulders, lumbar extensors, abdominals, biceps, triceps, back, and lower limbs. The exercises used nautilus machines, cable machines, and body weight.	**PIRE** Exercise intensity progressed from 40% to 60% 1RM (upper body) and 80% 1RM (lower body). When 12 proper reps were achieved, weight increased by 2.3–4.5 kg.
Valkeinen et al., 2005 [26]	RCT	RE: n = 13 GC: n = 13	RE: 60 ± 2 GC: 59 ± 4	12 weeks: WF: 2 week **Exercises:** The program included 6–7 isotonic exercises that are full-body exercises with a greater focus on the upper and lower extremities.	**PIRE** The program began at an intensity of 40% of 1RM and progressed to 80% of 1RM. Subjects kept an exercise diary to record the loads used.
Gavi et al., 2014 [38]	RCT	RE: n = 35 FG: n = 31	RE: 44.34 ± 7.94 FG: 48.65 ± 7.60	16 weeks: WF: 2 week **Exercises:** Supervised progressive weight training, 8 muscle groups (quads, hamstrings, biceps, triceps, pecs, calves, deltoids, and lats), 12 exercises, 3 × 12 reps.	**PIRE** RE progressed from 40% to 80% of 1RM over 15 weeks. Load increases are assessed every 3–4 weeks. A total of 42 participants (62.7%) reached 80% 1RM, and 7 (10.4%) reached 60% 1RM.
Larsson et al., 2015 [37]	RCT	RE: n = 67 GC: n = 63	RE: 50.81 ± 9.05 GC: 52.10 ± 9.78 años	15 weeks: WF: 2 week **Exercises:** The protocol included 12 isotonic resistance exercises targeting 8 major muscle groups using weight machines, free weights, and body weight. Key exercises included leg presses, knee extensions, biceps curls, and heel raises. Explosive strength exercises, like rapid heel raises and knee extensions, were added in the later weeks.	**PIRE** Weeks 1–2: 40% 1RM, 15–20 repetitions, 1–2 sets Weeks 3–5: 60% 1RM, 10–12 repetitions, 1–2 sets Weeks 6–15: 80% 1RM, 5–8 repetitions, 1–2 sets
Ericsson et al., 2016 [32]	RCT	RE: n = 67 RE: n = 63	RE: 50.81 ± 9.05 RE: 52.10 ± 9.78	15 weeks: WF: 2 week **Exercises:** The resistance exercise program targeted major muscle groups using a combination of weight machines and free weights, including leg press, knee extension/flexion, biceps curl, hand grip, heel raises, core stability, chest press, triceps extensions, and shoulder exercises.	**PIRE** Progressive, starting at 40% of 1RM and progressing up to 80% of 1RM.
Ernberg et al., 2016 [33]	RCT	RE: n= 24 GC: n = 27	>18	15 weeks: WF: 2 week Each session started with 10 min of bicycling to warm up and was then followed by 50 min of resistance exercise (major muscle groups).	**PIRE** The exercise was initiated at low loads at 40% of the maximum voluntary capacity (MVC) and successively progressed up to 70–80% of MVC.
Assumpção et al., 2018 [35]	RCT	RE: n = 16 FG: n = 14 GC: n = 14	RE: 45.7 ± 7.7 FG: 47.9 ± 5.3 GC: 46.9 ± 6.5	12 weeks: WF: 2 week **Exercises:** 40 min sessions: The stretching group did active stretches and isometrics; the resistance group used weights and isometric holds, both targeting full-body muscles.	**PIRE** Started without a load in the first two sessions Then, increased by 0.5 kg each week if the effort was perceived as “slightly intense” on the Borg scale. Progression was based on perceived effort, not on a percentage of 1RM. Performed 1 set of 8 repetitions for each exercise
Silva et al., 2019 [36]	**RCT**	RE: n = 30 RE: n = 30	GE: 49.40 ± 8.30 RE: 44.93 ± 10.30	12 weeks: WF: 2 week **Exercises:** The protocol consisted of resistance training targeting the upper body (arm, chest, and shoulder muscles), and lower extremity muscles (knee and hip musculature), performing 3 sets of 12 repetitions. The control group underwent relaxation sessions with music in a temperature-controlled environment.	**PIRE** StaREing intensity: 60% of 1RM in the first month Progressive increase: 70% of 1RM in the second month 80% of 1RM in the third month
Jablochkova et al., 2019 [34]	RCT	RE: n = 41 RE: n = 34 HC: n = 25	20–65	15 weeks: WF: 2 week **Exercises:** The resistance exercise group performed sessions twice a week, which included 10 min of warm-up followed by 50 min of strength training, focusing mainly on the lower limbs. Meanwhile, the relaxation group participated in 25 min sessions twice a week, consisting of guided relaxation therapy with mental exercises, relaxation, and autosuggestion, ending with stretching exercises.	**PIRE** Intensity progressed from an initial 40% of maximum voluntary capacity (MVC) to 70–80% of MVC.
Izquierdo-Alventosa et al., 2020 [30]	RCT	RE: n = 16 GC: n = 16	RE: 53.06 ± 8.4 GC: 55.13 ± 7.35	8 weeks: WF: 2 week **Exercises:** They performed 60 min resistance and coordination training sessions, including warm-up, main workout, and cool-down. The training involved walking and a circuit of 10 exercises targeting muscles of the upper and lower limbs: biceps, shoulders, pectorals, quadriceps, hip abductors, and calves. Each exercise was performed for 15 to 25 repetitions	**LIRE** Training intensity was adjusted using the Borg CR-10 scale. Initially, weak effort (1–2) was sought, later increasing to moderate (3–4). Weights of 0.5–2 kg for arms and 1–3 kg for legs were used, plus soft elastic bands. The intensity was adapted based on each participant’s pain and effort.
Andersson et al., 2021 [31]	RCT	RE (80% 1RM): n = 10 RE (50% 1RM): n = 10	RE: 22–46	2 to 3 weeks: WF: 2 week **Exercises:** The study utilized two resistance exercise protocols, each consisting of six main exercises including bench press, lunges, and squats.	**HIRE** The light/moderate load protocol was performed at 50% of 1RM with 20–30 repetitions, while the heavy load protocol was executed at 80% of 1RM with 7–8 repetitions. Both protocols were applied in separate sessions with a rest period between them.
Park et al., 2021 [27]	RCT	RE: 15 FG:15	RE: 52.8 ± 7.1 FG: 50.5 ± 7.1	4 weeks: WF: 2 week **Exercises:** The program combined core stabilization exercises (drawing-in maneuver, bridges, and bird dog) and strengthening movements (sit-ups with/without weights, crunches, and back extensions). The stretching protocol included 2 sets of 3 repetitions (30 s hold) for 8–9 exercises targeting pain areas	**MIRE** Initially perceived as moderate, with a score of 12.1 (Borg scale), corresponding to approximately 50–60% of maximum effort.
Arakaki et al., 2021 [39]	RCT	RE: n = 30 FG: n = 30	RE: 47.4 ± 9.0 FG: 47.3 ± 8.7	12 weeks: WF: 3 week **Exercises:** Swiss ball group: 8 strengthening exercises targeting major muscle groups, 3 sets of 12 repetitions Stretching group: Stretches targeting the same muscles as the Swiss ball group, 3 sets of 30 s per stretch	**MIRE** 60% de 1RM
Maestre-Cascales et al., 2022 [25]	EXP	RE: n = 41	RE: 56.36 ± 8.72	24 weeks: WF: 2 week **Exercises:** Three progressive phases: Free weights and bodyweight exercises (5 weeks) Added elastic bands (7 weeks) Added external loads (12 weeks) Exercises targeted upper and lower limb muscles and trunk muscles.	**PIRE** Weeks 1–5: 3–4 on the OMNI-GSE scale Weeks 6–12: 4–5 on the OMNI-GSE scale Weeks 13–24: 6–8 on the OMNI-GSE scale
Vilarino et al., 2022 [20]	RCT	RE (Low intensity): n = 9 RE (High intensity): n = 7 RE (Preferred intensity) n = 10 HC: n = 27	RE (Low intensity): n = 9 RE (High intensity): n = 7 RE (Preferred intensity): n = 10 HC: n = 27	8 weeks: WF: 2 week **Exercises:** Isotonic: standing calf raise, leg press, squat, low row, shoulder press, and bench press.	**PIRE** RE (Low intensity): 2 sets of 12 repetitions, 1 min rest between sets. RE (High intensity): 4 sets of 6 maximum repetitions, 2 min rest between sets RE (Preferred intensity): 3 sets of 8–12 repetitions based on tolerance, 1 min rest between sets

Note: RE = resistance exercise; EXP = experimental study; FG = flexibility group; HC = healthy control; CG = control group; WF = frequency per week; 1RM = one repetition maximum; OMNI-GSE = Generalized Subjective Effort; RCT = randomized controlled trial; HIRE = high-intensity resistance exercise; MIRE = medium-intensity resistance exercise; LIRE = low-intensity resistance exercise; PIRE = progressive-intensity resistance exercise.

**Table 3:** Results were analyzed in the selected studies post-intervention of RE at different intensities (HIRE, MIRE, LIRE, and PIRE) in patients with FM.

Authors	Intensity	Pain	Physical Functional Capacity	Depression	Sleep Quality	Quality Life
Häkkinen et al. [24]	PIRE	↑	↑	↑	-	-
Jones et al. [28]	LIRE	↑	-	↑	-	↔
Kingsley et al. [29]	PIRE	↔	↑	-	-	-
Valkeinen et al. [26]	PIRE	↔	-	-	-	-
Gavi et al. [38]	PIRE	↑	↑	↑	-	↑
Larsson et al. [37]	PIRE	↑	-	↑	↑	↑
Ericsson et al. [32]	PIRE	↔	-	↔	-	↔
Ernberg et al. [33]	PIRE	↑	-	↔	-	↑
Assumpção et al. [35]	PIRE	↑	↑	↑	-	↑
Silva et al. [36]	PIRE	↑	↑	↑	-	↑
Jablochkova et al. [34]	PIRE	↑	↑	↔	-	↔
Izquierdo-Alventosa et al. [30]	LIRE	↑	↑	↑	-	↑
Andersson et al. [31]	HIRE	↔	-	-	↑	-
Park et al. [27]	MIRE	↑	↑	↔	-	↔
Arakaki et al. [39]	MIRE	↑	↑	↑	-	↑
Maestre-Cascales et al. [25]	PIRE	↑	↑	↔	↑	-
Vilarino et al. [20]	PIRE	-	-	↔	-	-

Note: PIRE = progressive-intensity resistance exercise; LIRE = low-intensity resistance exercise; MIRE = medium-intensity resistance exercise; HIRE = high-intensity resistance exercise; ↑ = positive and significant effect; - = not studied; ↔ = no effect.

**Table 4:** Risk of bias assessment for parallel design studies using the Cochrane RoB 2 tool.

Study	D1: Randomization	D2: Deviations	D3: Missing Data	D4: Measurement	D5: Selection	Overall Risk
Izquierdo-Alventosa et al. (2020) [30]	Low	Low	Low	Low	Some concerns	Some concerns
Assumpção et al. (2018) [35]	Low	Low	Low	High	Low	High
Silva et al. (2019) [36]	Low	Low	Low	Low	Low	Low
Vilarino et al. (2022) [20]	Low	Low	Low	Low	Low	Low
Larsson et al. (2015) [37]	Low	Low	Low	Low	Low	Low
Häkkinen et al. (2001) [24]	Low	Low	Low	High	Low	High
Jones et al. (2002) [28]	Low	Low	Low	Low	Low	Low
Kingsley et al. (2005) [29]	Low	Low	Low	Low	Low	Low
Valkeinen et al. (2005) [26]	Some concerns	Low	Low	High	Low	High
Gavi et al. (2014) [38]	High	Some concerns	Some concerns	Low	Some concerns	High
Ericsson et al. (2016) [32]	Some concerns	Low	Low	Low	Some concerns	Some concerns
Ernberg et al. (2016) [33]	Some concerns	Low	Low	Low	Some concerns	Some concerns
Jablochkova et al. (2019) [34]	Low	Low	Low	Low	Some concerns	Some concerns
Park et al. (2021) [27]	Low	Low	Low	Low	High	High
Arakaki et al. (2021) [39]	Low	Low	Low	Low	High	High
Maestre-Cascales et al. (2022) [25]	High	Low	Low	Low	High	High

Legend: D1: randomization process; D2: deviations from intended interventions; D3: missing outcome data; D4: measurement of the outcome; D5: selection of the reported result.

**Table 5:** Risk of bias assessment for crossover design study using the adapted RoB 2 version.

Study	D1: Randomization	D2: Deviations	D3: Missing Data	D4: Measurement	D5: Selection	D6: Carry-Over Effects	D7: Wash-Out Period	Overall Risk
Andersson et al. (2021) [31]	Low	Low	Low	Low	Low	Low	Low	Low

Legend: D1: randomization process; D2: deviations from intended interventions; D3: missing outcome data; D4: measurement of the outcome; D5: selection of the reported result; D6: assessment of carry-over effects; D7: adequacy of wash-out period.

## Data Availability

No new data were created in this study. The data analyzed in this systematic review are available in the original articles cited in the References Section.

## Abbreviations

The following abbreviations are used in this manuscript:

1RM	one repetition maximum
BDI-FS	Beck Depression Inventory-Fast Screen
BPI	Brief Pain Inventory
BRUMS	Brunel Mood Scale
CG	control group
CS-PFP	Continuous-Scale Physical Functional Performance
FG	flexibility group
FIQ	Fibromyalgia Impact Questionnaire
FIQR	Fibromyalgia Impact Questionnaire-Revised
FM	fibromyalgia
HADS	Hospital Anxiety and Depression Scale
HAQ	Health Assessment Questionnaire
HC	healthy control
HIRE	high-intensity resistance exercise
LIRE	low-intensity resistance exercise
MIRE	medium-intensity resistance exercise
MVC	maximum voluntary capacity
OMNI-GSE	OMNI Generalized Subjective Exertion Scale
PIRE	progressive-intensity resistance exercise
PRISMA	Preferred Reporting Items for Systematic Reviews and Meta-Analyses
PROSPERO	International Prospective Register of Systematic Reviews
PSQI	Pittsburgh Sleep Quality Index
QOL	quality of life
QOLS	Quality of Life Scale
RCT	randomized controlled trial
RE	resistance exercise
SF-36	Short Form-36 Health Survey
STAI-S	State-Trait Anxiety Inventory
TP	tender point
VAS	Visual Analog Scale
WF	Weekly Frequency

## Appendix A

**Table A1:** Database search strategy.

**PubMed—14 August 2024**		
**Search**	**Query**	**Items Found**
#10	((Fibromyalgia [MeSH Terms]) OR (Fibromyalgia [Title/Abstract])) AND (((((Resistance Training [MeSH Terms]) OR (Resistance Training [Title/Abstract])) OR (Strength Training [MeSH Terms])) OR (Strength Training [Title/Abstract])) OR (Resistance Exercise [Title/Abstract]))	33
#9	((((Resistance Exercise [Title/Abstract]) OR (Strength Training [Title/Abstract])) OR (Strength Training [MeSH Terms])) OR (Resistance Training [Title/Abstract])) OR (Resistance Training [MeSH Terms])	7351
#8	Resistance Exercise [Title/Abstract]	2.130
#7	Strength Training [Title/Abstract]	1.827
#6	Strength Training [MeSH Terms]	5.065
#5	Resistance Training [Title/Abstract]	3.526
#4	Resistance Training [MeSH Terms]	5.065
#3	(Fibromyalgia*[MeSH Terms]) OR (Fibromyalgia*[Title/Abstract])	1.124
#2	Fibromyalgia*[Title/Abstract]	1.091
#1	Fibromyalgia*[MeSH Terms]	1.028
**Scopus—14 August 2024**		
**Search**	**Query**	**Items Found**
#6	(fibromyalgia) AND (resistance AND training OR strength AND training OR resistance AND exercise)	68
#5	(resistance AND training OR strength AND training OR resistance AND exercise)	15.403
#4	Resistance AND Exercise	21,266
#3	Strength AND Training	21.366
#2	Resistance AND Training	19.201
#1	Fibromyalgia	6969
**SciELO—14 August 2024**		
**Search**	**Query**	**Items Found**
#6	(fibromyalgia) AND (resistance AND training OR strength AND training OR resistance AND exercise)	1
#5	(resistance AND training OR strength AND training OR resistance AND exercise)	275
#4	Resistance AND Exercise	870
#3	Strength AND Training	1.208
#2	Resistance AND Training	1.047
#1	Fibromyalgia	394
**Web of Science—14 August 2024**		
**Search**	**Query**	**Items Found**
#6	(fibromyalgia) AND (resistance AND training OR strength AND training OR resistance AND exercise)	211
#5	(resistance AND training OR strength AND training OR resistance AND exercise)	133,093
#4	Resistance AND Exercise	41,022
#3	Strength AND Training	68,197
#2	Resistance AND Training	59,017
#1	Fibromyalgia	12,073
**Science Direct—14 August 2024**		
**Search**	**Query**	**Items Found**
#6	(fibromyalgia) AND (resistance AND training OR strength AND training OR resistance AND exercise)	88
#5	(resistance AND training OR strength AND training OR resistance AND exercise)	2576
#4	Resistance AND Exercise	4164
#3	Strength AND Training	9494
#2	Resistance AND Training	4014
#1	Fibromyalgia	1078
**PEDro—14 August 2024**		
**Search**	**Query**	**Items Found**
#6	(fibromyalgia) AND (resistance AND training OR strength AND training OR resistance AND exercise)	4
#5	(resistance AND training OR strength AND training OR resistance AND exercise)	1236
#4	Resistance AND Exercise	3.835
#3	Strength AND Training	5059
#2	Resistance AND Training	3.727
#1	Fibromyalgia	669
